# Prognostic Effect of Age in Resected Pancreatic Cancer Patients: A Propensity Score Matching Analysis

**DOI:** 10.3389/fonc.2022.789351

**Published:** 2022-03-31

**Authors:** Yaolin Xu, Yueming Zhang, Siyang Han, Dayong Jin, Xuefeng Xu, Tiantao Kuang, Wenchuan Wu, Dansong Wang, Wenhui Lou

**Affiliations:** Department of General Surgery, Zhongshan Hospital Fudan University, Shanghai, China

**Keywords:** pancreatic ductal adenocarcinoma, the elderly patients, radical resection, adjuvant chemotherapy, prognosis

## Abstract

**Background:**

While the elderly population account for an indispensable proportion in pancreatic ductal adenocarcinoma (PDAC), these patients are underrepresented in clinical trials. Whether surgery offered the same benefit for elderly patients as that for younger cohort and which factors affected long-term outcome of elderly population remained unclear.

**Aims:**

This study aims to evaluate long-term prognosis of elderly PDAC patients (≥70 years old) after surgery and to investigate potential prognostic factors.

**Methods:**

This retrospective study included PDAC patients receiving radical resection from January 2012 to July 2019 in Zhongshan Hospital Fudan University. Patients were divided into young (<70) and old groups (≥70). Propensity score matching (PSM) was conducted to eliminate the confounding factors. We investigated potential prognostic factors *via* Cox proportional hazards model and Kaplan–Meier estimator. Nomogram model and forest plot were constructed to illustrate the prognostic value of age.

**Results:**

A total of 552 PDAC patients who received radical resection were included in this research. Elderly patients showed poorer nutritional status and were less likely to received adjuvant treatment. After matching, although age [hazard ratio (HR)=1.025, 95%CI 0.997–1.054; p=0.083] was not statistically significant in the multivariate cox regression analysis, further survival analysis showed that patients in the old group had poorer overall survival (OS) when compared with young group (p=0.039). Furthermore, reception of adjuvant chemotherapy (HR=0.411, 95%CI 0.201-0.837; p=0.014) was the only independent prognostic factor among elderly patients and could significantly improve OS. Subgroup analysis indicated that age had better prognostic value in PDAC patients with good preoperative nutritional status and relative low tumor burden. Finally, a prognostic prediction model contained age, reception of adjuvant chemotherapy, American Joint Committee on Cancer (AJCC) 8th T and N stage was constructed and presented in nomogram, whose Harrell’s concordance index was 0.7478 (95%CI, 0.6960–0.7996). The calibration curves at 1 and 3 years indicated an optimal conformity between actual and nomogram-predicted survival probability in the PDAC patient who received surgery.

**Conclusion:**

The elderly PDAC patients were associated with worse OS survival after radical resection, and the noticeable negative effect of age was observed among PDAC patients with better preoperative nutritional status and less aggressive tumor biology. Adjuvant chemotherapy was essential to improve survival outcome of elderly PDAC patients following radical resection.

## Introduction

Pancreatic adenocarcinoma (PDAC) is one of the most malignant solid tumors with poor prognosis. PDAC is strongly age dependent, and increasing population longevity and aging will contribute to the global burden of pancreatic cancer (1). A research derived from the Global Burden of Disease Study showed that incidence rate at ages older than 70 years was three to four times higher than those at ages 50–69 years in 2017, and 20.2% of these were attributable to population aging (2). By 2030, approximately 70% of PDAC will be diagnosed in older adults (3). While the elderly population accounted for an indispensable proportion on PDAC, the older cancer patients were underrepresented in clinical trials (4). A research demonstrated that patients aged 70 years or older accounted for the most of the under-representation among those noted in registration trials for all cancer treatment (5). A research involving of 10,505 PDAC patients based on Surveillance, Epidemiology, and End Results (SEER) database showed that over half of older patients (≥65) with potentially treatable pancreatic cancer did not receive any treatment at all. Only 11% of older patients with locoregional pancreatic cancer received multimodality therapy (6). Thus, the treatment strategies concluded from younger patients may not be implied into the very elderly patients completely. The optimal therapeutic strategy for the elderly patients with PDAC remained to be determined.

Currently, surgery was still the only treatment that could offer a chance to cure pancreatic cancer (7). However, it was uncertain whether surgery will benefit the elderly patients. Some researchers suggested that the prognosis in the elderly was poorer than in the younger patients (8, 9), while others hold the opposed points of view (10–12). What seems clear, though, is that the incidence of postoperative complications is much higher in the elderly patients (13–16). This could be one of the factors that influence the decisions of therapeutic strategies for the elderly patients. As a result, more clinical studies focused on the elderly should be performed to determine the optimal therapeutic strategy.

In the present research, we evaluated the postoperative long-term prognosis of the elderly patients (≥70) with PDAC by comparing with the younger patients. Moreover, we analyzed the prognostic factors for long-term survival in order to explore the optimal therapeutic strategies for the elderly patients.

## Materials and Methods

### Patients and Study Design

The research included PDAC patients receiving radical resection in Zhongshan Hospital Fudan University from January 2012 to July 2019. The inclusion criteria are as follows: (1) with definite pathological diagnosis of pancreatic adenocarcinoma; (2) with definite American Joint Committee on Cancer (AJCC) 8th TNM stage; (3) with complete preoperative blood samples test and all the tests were performed 1 week before surgery; (4) with sufficient follow-up time at least 24 months. The total cohort was divided into two groups included the young group (<70 years) and the old group (≥70 years), according to the age of patients. Overall survival (OS) was calculated from the date of surgery to the date of death or last follow-up. Patients were generally seen in follow-up 4–6 weeks following discharge and every 3–6 months thereafter for physical examination, laboratory test, and imaging to assess for postoperative recovery and cancer recurrence. Besides, telephone interviews every 3 months were performed to supplement follow-up information. Median follow-up time was 40 months. The study outcomes were overall survival (OS) from time of surgery. All the medical information and time of survival were obtained from medical records and telephone interviews.

### Clinicopathological Data

Patients’ demographic characteristics, pathological results, and blood sample results were extracted from electric medical records. Among the patients who received adjuvant chemotherapy and radiotherapy, only one patient accepted neoadjuvant chemotherapy. The adjuvant chemotherapy regimens were prescribed based on the latest clinical practice guidelines and the clinical evaluation of doctors. The most common chemotherapy regimens were combination regimens, including AS (albumin-bound paclitaxel + S-1), AG (albumin-bound paclitaxel + gemcitabine), GS (gemcitabine + S-1), and FOLFIRINOX. The patients with severe side effects or with negative intentions for chemotherapy mostly received single drug such as gemcitabine or S-1. Second-line treatment mainly depends on the clinical evaluation of doctors. The information of tumor location, AJCC 8th TNM stage, tumor differentiation, microvascular invasion, fatty invasion, and perineural invasion were defined by the pathological results. None of the study population was diagnosed with metastatic tumor. A carbohydrate antigen 19-9 (CA 19-9) cutpoint of 35 U/ml was used to dichotomize patients with normal and elevated values based on a research performed by Aldakkak and colleagues. Elevated CA 19-9 were then further stratified into low (36–200), moderate (201–1,000), and high (>1,000) groups (17). The American Society of Anesthesiologists Physical Status (ASA PS) classification system was performed by the anesthetists before surgery. The ASA classification system contains categories 1–5 and represents increasing levels of patient impairment (18).

### Statistical Analysis

Data analyses were performed by R project 3.5.3 for Windows and IBM SPSS Statistics 22.0 version. Normality and homogeneity of variance were tested by Shapiro–Wilk test and Levene’s test. Categorical variables were reported as frequencies and percentages. Continuous variables conforming to normal distribution were presented by means and standard error; others were described as medians and interquartile range. The baseline characteristics between different groups were compared using Fisher’s precision probability test for categorical variables, using Wilcoxon rank sum test for continuous variables, respectively. Propensity score matching was performed with “MatchIt” packages using R project. A 1:1 ratio propensity score matching study group was created using the nearest neighbor matching method with a 0.6 caliper. Survival curves were drawn with the method of Kaplan–Meier, and log-rank test was used to compare the overall survival of different groups. Cox proportional hazards model was used to estimate the hazard ratio of death. The significant statistical variables (p<0.1) in univariate Cox regression analysis were incorporated into the multivariate analysis to identify the independent prognostic factors for survival. Forest plot was performed to show the outcome of subgroup analysis. Forest plot was performed by “forestplot” packages using R project. The survival nomogram was developed starting from Cox model, which allowed to obtain survival probability estimates. The endpoints in building the nomogram model were 1- and 3-year survival. Harrell’s concordance index (C-index) was used in the nomogram to evaluate the model performance for predicting outcomes. The nomogram was subjected to 1,000 bootstrap resamples for internal validation of the cohort. Then, calibration curves were used to verify the accuracy between predicted and actual 1- and 3-year survival. All the significance tests in this paper were two-sided tests.

## Results

### Baseline Characteristics of the Total Cohort

A total of 552 patients diagnosed with PDAC who accepted radical surgery were incorporated in the total cohort. The patients aged 70 years or older were defined as the elderly in this study. The patients’ clinicopathological characteristics are listed in **Table 1**. There were 411 patients younger than 70 years old (young group) and 141 patients aged 70 years or older (old group). In the total cohort, the old group was less likely to receive adjuvant chemotherapy and radiotherapy and presented with earlier N stage. Besides, the prealbumin, albumin, white cell count, lymphocyte count, and AFP in the old group were significantly lower than that of the young group. Other factors did not differ significantly between two groups.

**Table 1:** Baseline characteristics of the study population (n=552).

	Total	Age<70 (n=411)	Age≥70 (141)	p-value
**Age**				
Median (IQR)	64.00 (58.00–70.00)	61.00 (56.00–65.00)	73.00 (72.00–76.00)	**< 0.001**
**Sex**				
Male	326 (59%)	243 (59%)	83 (59%)	1
Female	226 (41%)	168 (41%)	58 (41%)	
**Tumor location**				
Head	296 (54%)	227 (55%)	69 (49%)	0.27
Body and tail	243 (44%)	173 (42%)	70 (50%)	
Total pancreas	13 (2%)	11 (3%)	2 (1%)	
**CA19-9 level**				
<35	147 (27%)	113 (27%)	34 (24%)	0.7
35-200	195 (35%)	145 (35%)	50 (35%)	
>200	210 (38%)	153 (37%)	57 (40%)	
**Adjuvant chemotherapy**				
No	112 (21%)	69 (17%)	43 (32%)	**<0.001**
Yes	427 (79%)	334 (83%)	93 (68%)	
**Adjuvant radiotherapy**				
No	424 (77%)	306 (75%)	118 (86%)	**0.009**
Yes	124 (23%)	104 (25%)	20 (14%)	
**AJCC 8th T stage**				
T1	113 (21%)	84 (20%)	29 (21%)	0.37
T2	306 (56%)	223 (54%)	83 (59%)	
T3	84 (15%)	69 (17%)	15 (11%)	
T4	20 (4%)	16 (4%)	4 (3%)	
Tis	27 (5%)	18 (4%)	9 (6%)	
**AJCC 8th N stage**				
N0	317 (58%)	223 (55%)	94 (67%)	**0.036**
N1	193 (35%)	152 (37%)	41 (29%)	
N2	39 (7%)	33 (8%)	6 (4%)	
**Tumor differentiation**				
Well-diff	34 (6%)	23 (6%)	11 (8%)	0.06
Moderately-diff	216 (40%)	150 (37%)	66 (47%)	
Poorly-diff	294 (54%)	231 (57%)	63 (45%)	
**MVI**				
No	457 (83%)	335 (82%)	122 (87%)	0.2
Yes	95 (17%)	76 (18%)	19 (13%)	
**FI**				
No	131 (24%)	93 (23%)	38 (27%)	0.3
Yes	421 (76%)	318 (77%)	103 (73%)	
**NI**				
No	112 (20%)	88 (21%)	24 (17%)	0.28
Yes	440 (80%)	323 (79%)	117 (83%)	
**Preglucose**				
Median (IQR)	5.80 (5.10–6.90)	5.80 (5.10–6.80)	5.70 (5.10–7.20)	0.89
**Albumin**				
Median (IQR)	40.00 (38.00–43.00)	41.00 (39.00–43.00)	40.00 (38.00–42.00)	**<0.001**
**Prealbumin**				
Median (IQR)	0.23 (0.18–0.26)	0.23 (0.19–0.27)	0.21 (0.18–0.25)	**0.012**
**Hemoglobin**				
Median (IQR)	128.00 (118.50–139.00)	128.00 (120.00–139.00)	126.00 (116.00–136.00)	0.086
**WBC**				
Median (IQR)	5.34 (4.54–6.39)	5.42 (4.62–6.48)	5.20 (4.46–6.10)	**0.034**
**Neutrophil count**				
Median (IQR)	3.20 (2.40–3.90)	3.20 (2.50–3.90)	3.00 (2.40–3.90)	0.36
**Lymphocyte count**				
Median (IQR)	1.50 (1.20–1.90)	1.50 (1.30–1.90)	1.40 (1.10–1.70)	**0.003**
**Monocyte count**				
Median (IQR)	0.42 (0.34–0.53)	0.42 (0.34–0.52)	0.43 (0.37–0.54)	0.28
**ASA**				
Grade 1	71 (14.95%)	56 (15.77%)	15 (12.50%)	0.39
Grade 2	391 (82.32%)	291 (81.97%)	100 (83.33%)	
Grade 3	13 (2.74%)	8 (2.25%)	5 (4.17%)	
**AFP**				
Median (IQR)	2.60 (1.90–3.60)	2.70 (2.00–3.70)	2.40 (1.60–3.30)	**0.015**
**CEA**				
Median (IQR)	3.00 (1.80–4.80)	2.90 (1.75–4.70)	3.30 (2.20–5.20)	0.12
**CA242**				
Median (IQR)	22.95 (8.88–70.48)	23.30 (8.60–62.70)	22.20(9.65–121.50)	0.49
**CA50**				
Median (IQR)	68.80 (20.30–180.00)	66.10 (17.45–180.00)	83.80 (29.30–180.00)	0.12
**CA125**				
Median (IQR)	14.25 (9.40–23.73)	14.50 (9.50–24.00)	13.50 (9.00–22.70)	0.36

IQR, interquartile range; CA, carbohydrate antigen; AJCC, American Joint Committee on Cancer; Tis, tumor in situ; MVI, microvascular invasion; FI, peripancreatic fat invasion; NI, neural invasion; WBC, white blood cell; ASA, American Society of Anesthesiologists; AFP, alpha-fetoprotein; CEA, carcinoembryonic antigen. The bold values indicates statistically significance.

The univariate and multivariate Cox regression analysis demonstrated that age was not an independent prognostic factor [hazard ratio (HR)=1.005; 95% confidential index (CI), 0.993–1.018; p=0.416, **Supplementary Table S1**]. Further survival analyses showed no survival difference between two groups among all PDAC patients [old vs. young, median OS (mOS), 29.2 vs. 28.5 months, p=0.82, **Supplementary Figure S1**].

### Propensity Score Matching and Survival Analysis

In order to balance confounding factors that might affect survival outcome, propensity score matching (PSM) was performed by a 1:1 ratio. The potentially adjusting variables were based on the results of Cox regression analysis, which included AJCC 8th T stage (p=0.062), N stage (p=0.05), adjuvant chemotherapy (p<0.001), and CA50 (p=0.034). A total of 95 patients younger than 70 years old were matched with 95 patients older than 70 years old in the total cohort. The baseline characteristics between the two groups after PSM are listed in **Table 2**. All adjusting variables were comparable after PSM.

**Table 2:** Baseline characteristics of the study population after PSM (n=190).

	Total	Age<70 (n=95)	Age≥70 (n=95)	p-value
**Age**				
Median (IQR)	69.50 (62.25–73.00)	62.00 (57.50–66.50)	73.00 (72.00–76.00)	**<0.001**
**Sex**				
Male	113 (59%)	54 (57%)	59 (62%)	0.55
Female	77 (41%)	41 (43%)	36 (38%)	
**Tumor location**				
Head	87 (46%)	44 (46%)	43 (45%)	0.93
Body and tail	98 (52%)	48 (51%)	50 (53%)	
Total pancreas	5 (3%)	3 (3%)	2 (2%)	
**CA19-9**				
<35	41 (22%)	20 (21%)	21 (22%)	0.83
35–200	70 (37%)	37 (39%)	33 (35%)	
>200	79 (42%)	38 (40%)	41 (43%)	
**Adjuvant chemotherapy**				
No	57 (30%)	25 (26%)	32 (34%)	0.34
Yes	133 (70%)	70 (74%)	63 (66%)	
**Adjuvant radiotherapy**				
No	153 (81%)	71 (75%)	82 (86%)	0.066
Yes	37 (19%)	24 (25%)	13 (14%)	
**AJCC 8th T stage**				
T1	41 (22%)	22 (23%)	19 (20%)	0.85
T2	107 (56%)	50 (53%)	57 (60%)	
T3	20 (11%)	11 (12%)	9 (9%)	
T4	8 (4%)	5 (5%)	3 (3%)	
Tis	14 (7%)	7 (7%)	7 (7%)	
**AJCC 8th N stage**				
N0	124 (65%)	62 (65%)	62 (65%)	0.76
N1	60 (32%)	31 (33%)	29 (31%)	
N2	6 (3%)	2 (2%)	4 (4%)	
**Tumor differentiation**				
Well-diff	15 (8%)	7 (8%)	8 (9%)	0.88
Moderately diff	86 (46%)	41 (45%)	45 (48%)	
Poorly diff	84 (45%)	43 (47%)	41 (44%)	
**MVI**				
No	159 (84%)	80 (84%)	79 (83%)	1
Yes	31 (16%)	15 (16%)	16 (17%)	
**FI**				
No	49 (26%)	26 (27%)	23 (24%)	0.74
Yes	141 (74%)	69 (73%)	72 (76%)	
**NI**				
No	39 (21%)	22 (23%)	17 (18%)	0.47
Yes	151 (79%)	73 (77%)	78 (82%)	
**Preglucose**				
Median (IQR)	5.80 (5.00–6.80)	5.80 (5.00–6.50)	5.80 (5.00–7.10)	0.82
**Albumin**				
Median (IQR)	40.00 (38.00–42.00)	41.00 (39.00–43.00)	40.00 (37.00–42.00)	**<0.001**
**Prealbumin**				
Median (IQR)	0.22 (0.19–0.25)	0.23 (0.20–0.26)	0.21 (0.18–0.24)	**0.006**
**Hb**				
Median (IQR)	126.50 (116.00–138.00)	126.00(116.00–139.00)	128.00(115.25–136.75)	0.86
**WBC**				
Median (IQR)	5.34 (4.48–6.29)	5.52 (4.53–6.42)	5.21 (4.46–6.11)	0.24
**Neutrophil count**				
Median (IQR)	3.20 (2.50–3.90)	3.25 (2.62–3.80)	3.05 (2.42–3.98)	0.66
**Lymphocyte count**				
Median (IQR)	1.50 (1.20–1.80)	1.50 (1.30–1.90)	1.40 (1.10–1.70)	0.14
**Monocyte count**				
Median (IQR)	0.43 (0.35–0.54)	0.43 (0.34–0.54)	0.44 (0.37–0.55)	0.26
**ASA**				
Grade 1	29 (16%)	16 (18%)	13 (14%)	0.79
Grade 2	143 (80%)	70 (79%)	73 (81%)	
Grade 3	7 (4%)	3 (3%)	4 (4%)	
**AFP**				
Median (IQR)	2.40 (1.67–3.40)	2.60 (1.80–3.48)	2.35 (1.50–3.18)	0.15
**CEA**				
Median (IQR)	3.10 (1.90–4.80)	3.00 (1.70–4.70)	3.30 (2.20–5.52)	0.17
**CA242**				
Median (IQR)	30.00 (11.65–120.90)	33.80 (14.00–86.15)	26.70 (10.30–141.40)	0.58
**CA50**				
Median (IQR)	84.75 (30.45–180.00)	88.50 (33.25–180.00)	75.20 (29.30–180.00)	0.79
**CA125**				
Median (IQR)	13.70 (8.95–22.55)	12.80 (8.50–20.50)	14.25 (9.38–24.15)	0.21

IQR, interquartile range; CA, carbohydrate antigen; AJCC, American Joint Committee on Cancer; Tis, tumor in situ; MVI, microvascular invasion; FI, peripancreatic fat invasion; NI, neural invasion; WBC, white blood cell; ASA, American Society of Anesthesiologists; AFP, alpha-fetoprotein; CEA, carcinoembryonic antigen. The bold values indicates statistically significance.

**Figure 1:**
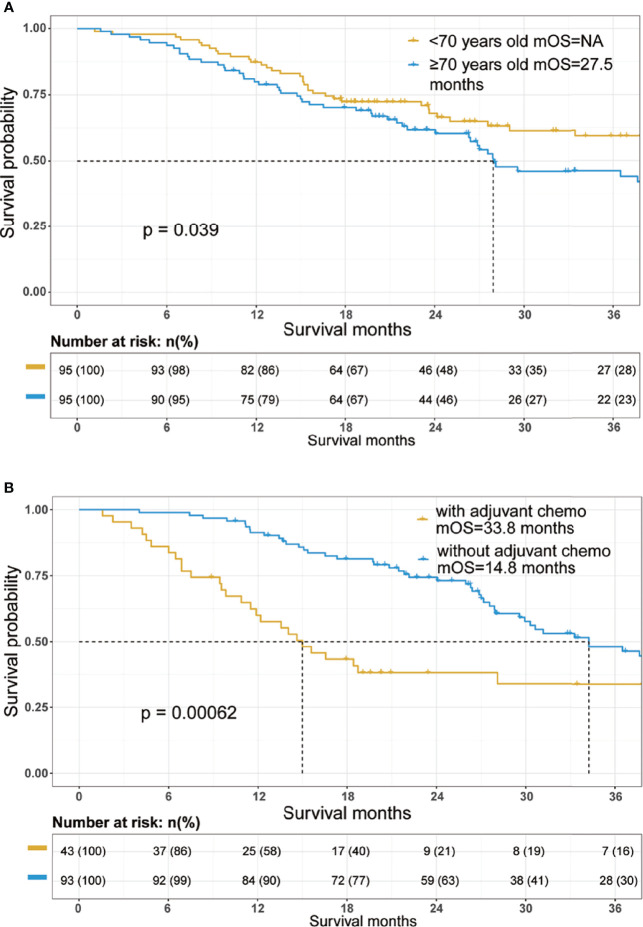
Overall survival Kaplan–Meier survival curves of the cohort. **(A)** Overall survival curves stratified by age in the total cohort after propensity score matching. **(B)** Overall survival curves stratified by the reception of adjuvant chemotherapy in the patients aged 70 years and older. mOS, median overall survival.

Cox proportional hazards models were constructed to investigate potential prognostic factors in matching cohort (**Table 3**). Univariate Cox regression analysis indicated that age, reception of chemotherapy, AJCC 8th T and N stage, peripancreatic fat invasion, perineural invasion, CA 19-9 level, albumin, and CA50 were independent prognostic factors. These factors were then incorporated into multivariate Cox regression analysis. As shown in **Table 3**, the reception of chemotherapy (HR=0.291; 95%CI, 0.173–0.287; p<0.001) and AJCC 8th T stage (T4, HR=3.706; 95%CI, 1.373–10.002; p=0.01) were independent prognostic factors, although age (HR=1.025; 95%CI, 0.997–1.054; p=0.083) was not statistically significant in the multivariate Cox regression analysis. Further survival analysis showed that patients in the old group had poorer OS when compared with the young group (old vs. young, mOS, 27.5 vs. NA months, p=0.039, **Figure 1A**).

**Table 3:** Univariate and multivariate Cox regression analysis of the cohort after PSM.

	Total cohort			
	Univariate		Multivariate	
	HR	p-value	HR	p-value
**Age (ref=age<70)**	1.043(1.017–1.069)	**0.001**	1.025(0.997–1.054)	**0.083**
**Gender (ref=male)**	0.834(0.539–1.290)	0.414		
**Tumor location (ref=pancreatic head)**		0.415		
**pancreatic body/tail**	0.752(0.490–1.155)	0.194		
**total pancreas**	0.724(0.175–2.987)	0.655		
**AJCC 8th T stage (ref=T1)**		**0.007**		**0.011**
**T2**	1.777(0.969–3.259)	0.063	1.425(0.753–2.697)	0.277
**T3**	2.459(1.117–5.412)	0.025	1.706(0.743–3.920)	0.208
**T4**	3.534(1.339–9.326)	0.011	3.706(1.373–10.002)	0.01
**Tis**	0.157(0.020–1.199)	0.074	0.088(0.010–0.801)	0.031
**AJCC 8th N stage (ref=N0)**		**0.039**		0.182
**N1**	1.489(0.946–2.343)	0.086	1.292(0.799–2.091)	0.296
**N2**	2.793(1.105–7.061)	0.03	2.309(0.877–6.081)	0.09
**Differentiation (ref=moderately diff)**		0.107		
**Poorly diff**	10.831(1.482–79.135)	0.019		
**Moderately diff**	12.021(1.653–87.409)	0.014		
**Un-diff**		0.977		
**MVI (ref=no MVI)**	1.214(0.682–2.161)	0.51		
**FI (ref=no FI)**	2.239(1.259–3.980)	**0.006**	1.164(0.619–2.191)	0.637
**NI (ref=no NI)**	2.193(1.186–4.056)	**0.012**	0.865(0.433–1.728)	0.682
**Adjuvant chemotherapy (ref=no-chemo)**	0.456(0.294–0.705)	**<0.001**	0.291(0.173–0.487)	**<0.001**
**Adjuvant radiotherapy (ref=no-radio)**	1.286(0.776–2.129)	0.329		
**CA19-9 level (ref=CA19-9<35)**		**0.044**		0.683
**CA19-9 35-200**	1.416(0.738–2.716)	0.295	1.414(0.642–3.114)	0.39
**CA19-9>200**	2.078(1.117–3.867)	0.021	1.652(0.454–6.012)	0.447
**Preglucose (continuous)**	1.016(0.914–1.129)	0.768		
**Albumin (continuous)**	0.945(0.892–1.001)	**0.056**	1.013(0.950–1.080)	0.703
**Prealbumin (continuous)**	0.064(0.001–3.060)	0.163		
**Hemoglobin (continuous)**	0.996(0.983–1.009)	0.524		
**WBC (continuous)**	0.894(0.773–1.035)	0.135		
**Lymphocyte count (continuous)**	0.759(0.513–1.123)	0.167		
**Neutrophil count (continuous)**	0.894(0.744–1.075)	0.234		
**Monocyte count (continuous)**	1.249(0.318–4.905)	0.75		
**ASA (ref=grade1)**		0.512		
**Grade 2**	0.811(0.461–1.428)	0.468		
**Grade 3**	0.443(0.101–1.941)	0.28		
**AFP (continuous)**	0.886(0.756–1.038)	0.133		
**CEA (continuous)**	1.009(0.992–1.026)	0.294		
**CA242 (continuous)**	1.004(0.999–1.008)	0.095		
**CA50 (continuous)**	1.004(1.001–1.007)	**0.016**	1.001(0.994–1.008)	0.772
**CA125 (continuous)**	1.003(0.997–1.008)	0.322		

HR, hazard ratio; ref, reference; CA, carbohydrate antigen; AJCC, American Joint Committee on Cancer; Tis, tumor in situ; MVI, microvascular invasion; FI, peripancreatic fat invasion; NI, neural invasion; WBC, white blood cell; ASA, American Society of Anesthesiologists; AFP, alpha-fetoprotein; CEA, carcinoembryonic antigen. The bold values indicates statistically significance.

The 1-year survival rate was 87% in the young group and 79% in the old group. The 3-year survival rate was 59% in the young group and 44% in the old group.

We further investigated potential prognostic factors in patients aged 70 and over using Cox regression analysis. As shown in **Table 3**, reception of chemotherapy (HR=0.411; 95%CI, 0.201–0.837; p=0.014) was the only independent prognostic factor in elderly PDAC patients who received surgery. The survival analysis further confirmed that adjuvant chemotherapy significantly improved OS among elderly PDAC patients (no adjuvant chemotherapy vs. receiving adjuvant chemotherapy, mOS, 14.8 vs. 33.8 months, p=0.00062, **Figure 1B**).

### Prognostic Nomogram Development and Validation

**Figure 2:**
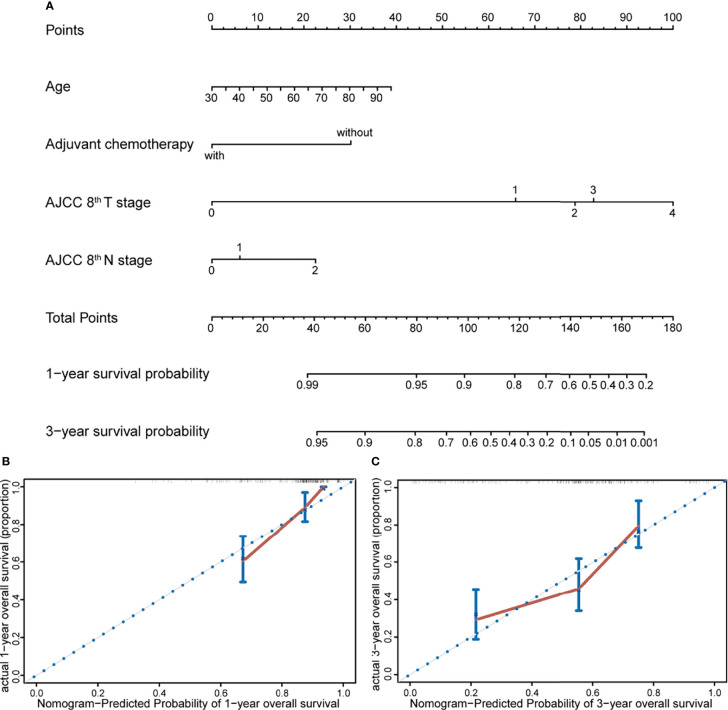
Nomogram and calibration plot for prediction of 1- and 3-year survival. **(A)** Prognostic nomogram for predicting survival probabilities of PDAC patients who received radical resection, constructed by age, reception of adjuvant chemotherapy, and AJCC 8th T and N stage. The 1- and 3-year survival probability could be predicted according to the total points, which were calculated by the summation of each factor’s points from the points scale. **(B, C)** Calibration plot of the prognostic nomogram for 1- and 3-year survival, respectively. The nomogram was subjected to 1,000 bootstrap resamples for internal validation of the cohort, and the accuracy of this model could be demonstrated by comparing the actual and predicted probabilities of overall survival.

A prognostic nomogram model was constructed according to the results of multivariate Cox regression analysis (**Figure 2A**). The prediction model incorporated independent prognostic factors in the Cox analysis including age, reception of chemotherapy, and AJCC 8th T and N stage. Each factor could get a point based on their grade from the points scale. The 1- and 3-year survival probability could be predicted according to the total points. The Harrell’s concordance index of this model was 0.7478 (95%CI, 0.6960–0.7996). Then, the nomogram model was subjected to 1,000 bootstrap resamples for internal validation of the cohort. The calibration curves at 1 and 3 years indicated an optimal conformity between actual and nomogram-predicted survival probability in the PDAC patient received surgery (**Figures 2B, C**).

### Subgroup Analysis of the Cohort After PSM

**Figure 3:**
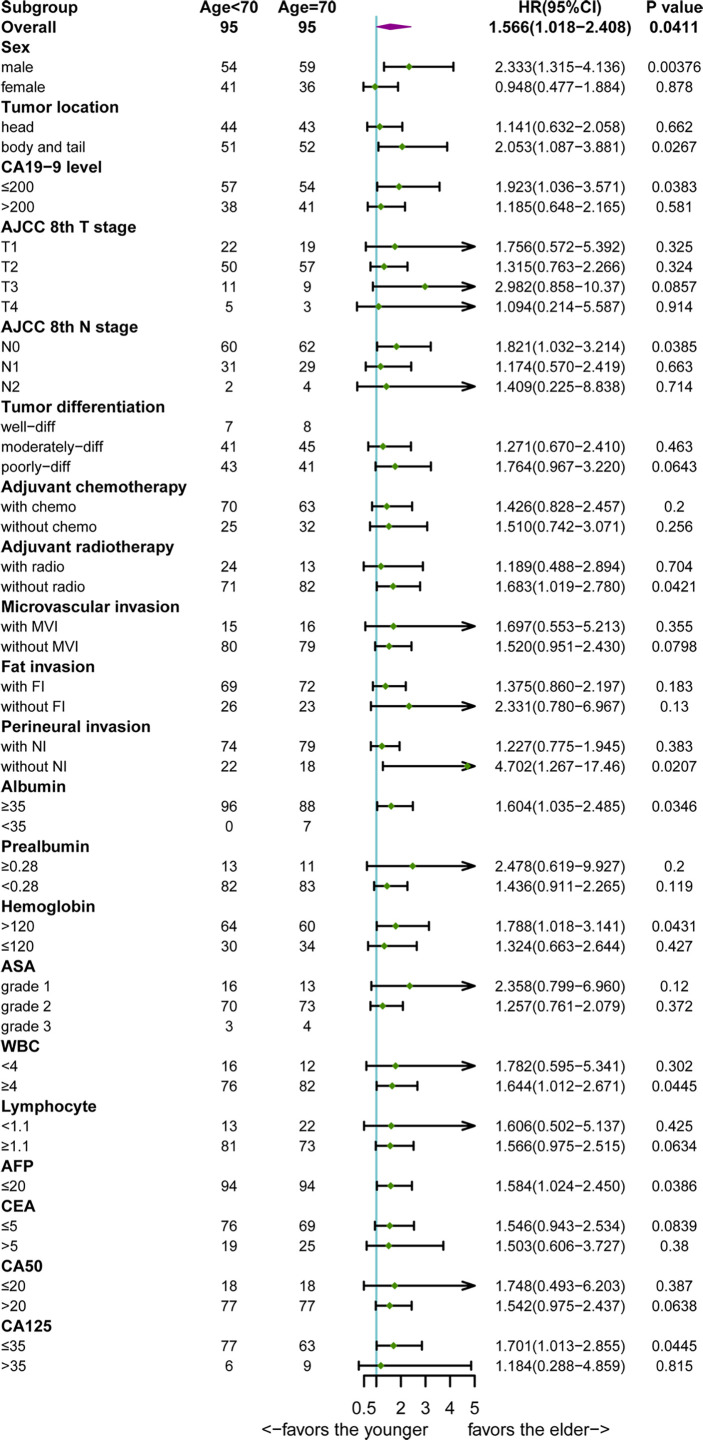
Forest plot of overall survival hazard ratios (HRs) of major subgroups in the cohort after propensity score matching.

**Figure 4:**
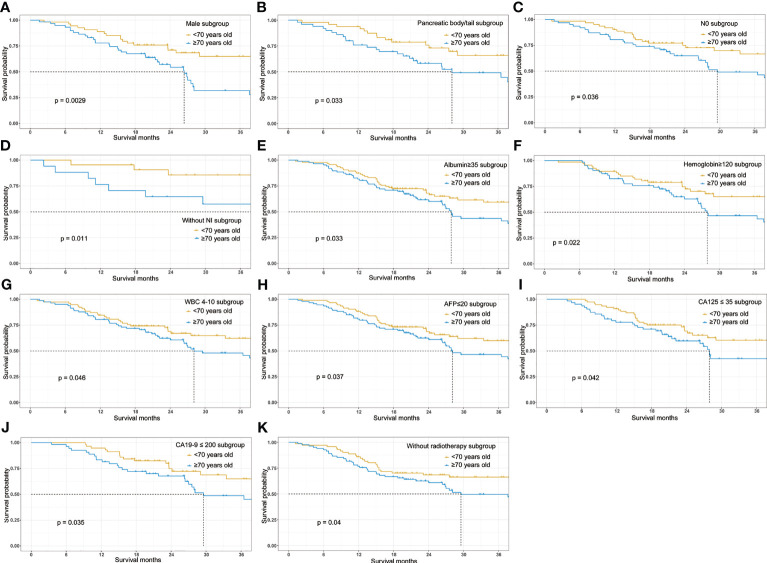
Overall survival Kaplan-Meier survival curves stratified by age (≥70 vs <70) in the major subgroups. **(A)** Survival curve stratified by age in male patients. **(B)** Survival curve stratified by age in patients with pancreatic body/tail cancer. **(C)** Survival curve stratified by age in patients without lymphatic metastasis. **(D)** Survival curve stratified by age in patients without perineural invasion. **(E)** Survival curve stratified by age in patients with serum albumin higher than 35g/L. **(F)** Survival curve stratified by age in patients with hemoglobin higher than 120g/L. **(G)** Survival curve stratified by age in patients with white cell count (WBC) between 4 and 10×109/L. **(H)** Survival curve stratified by age in patients with AFP lower than 20ng/ml. **(I)** Survival curve stratified by age in patients with CA125 lower than 35 ng/ml. **(J)** Survival curve stratified by age in patients with CA19-9 lower than 200U/ml. **(K)** Survival curve stratified by age in patients who didn’t receive adjuvant radiotherapy following surgery.

Further subgroup analyses were conducted to explore whether age remained as a prognostic factor in a certain subgroup. Forest plot (**Figure 3**) showed that the elderly may have poorer prognosis in male (HR=2.333; 95%CI, 1.315–4.136; p=0.00376) patients whose tumor was located at the pancreatic body/tail (HR=2.053; 95%CI, 1.087–3.881; p=0.0267), with N0 stage (HR=1.821; 95%CI, 1.032–3.214; p=0.0385), without perineural invasion (HR=4.702; 95%CI, 1.267–17.46; p=0.0207), albumin higher than 35 g/L (HR=1.604; 95%CI, 1.035–2.485; p=0.0346), hemoglobin higher than 120 g/L (HR=1.788; 95%CI, 1.018–3.141; p=0.0431), white blood cell count between 4 and 10×10^9^/L (HR=1.644; 95%CI, 1.012–2.671; p=0.0445), AFP lower than 20 ng/ml (HR=1.584; 95%CI, 1.024–2.450; p=0.0386), CA125 lower than 35 ng/ml (HR=1.701; 95%CI, 1.013–2.855; p=0.0445), CA19-9 lower than 200 U/ml (HR=1.923; 95%CI, 1.036–3.571; p=0.0383), and those patients who did not receive radiotherapy (HR=1.683; 95%CI, 1.019–2.780; p=0.0421). The survival curves between the young and the old group were compared using log-rank method and indicated the prognostic effect of age in these subgroups (**Figure 4**).

## Discussion

In the present research, we investigated the prognostic value of age in PDAC patients following radical resection. A total of 552 PDAC patients who received radical resection were included in this research. The elderly showed poorer preoperative nutritional status but earlier N stage and were less likely to receive adjuvant treatment. PSM was then conducted to eliminate the selected bias. After matching, although age (HR=1.025; 95%CI, 0.997–1.054; p=0.083) was not statistically significant in the multivariate Cox regression analysis, further survival analysis showed that patients in the old group had poorer OS when compared with the young group (old vs. young, mOS, 27.5 vs. NA months, p=0.039, **Figure 1A**). Furthermore, we found that reception of adjuvant chemotherapy was the only protective factor in the elderly patients. Subgroup analysis indicated that age had better prognostic value in resected PDAC patients with good preoperative nutritional status and relative low tumor burden.

At present, there is controversy about whether age affected the prognosis of PDAC patients following radical resection. Several studies suggested that age was not an independent prognostic factor, and there were no significant differences in OS between younger and older patients (19–21). On the other hand, some researchers hold the opposed points that the very elderly patients had poorer prognosis after surgery. A research retrospectively included 148,080 periampullary cancer patients, and they demonstrated that age was a factor attenuating the survival of patients with resected periampullary cancers (p<0.001). Besides, their research indicated that octogenarian patients who received radical resection showed superior long-term survival than those who did not undergo surgical treatment (22). Another research that incorporated 1,271 patients who received pancreaticoduodenectomy showed that patients older than 70 years had significantly shorter long-term survival (3-year survival, 0% vs. 29%, p<0.0001) (23). A retrospective multicenter analysis demonstrated that the prognosis of octogenarians was poorer than that of younger patients for both resectable and borderline resectable tumors (median survival time, 16.6 vs. 23.2 months, p=0.006) (24). However, the baseline characteristics between the very elderly and younger patients were unbalanced in most studies. Baseline imbalance in factors that are strongly related to outcome measures could cause bias in effect estimate. Thus, in our research, Cox proportional hazard model was constructed to investigate potential prognostic factors, and PSM was conducted to balance baseline characteristics that related to survival outcome. After matching, although age was not an independent prognostic factor in the multivariate Cox regression analysis, further survival analysis showed that patients in the old group had poorer OS when compared with the young group. While previous researchers hold the view that worse survival outcome in the elderly owed to low proportion of receiving adjuvant chemotherapy and poor preoperative nutritional status, we believed the survival disadvantage may be attribute to biological aging, which could lead to vulnerability to cancer and increase risk of cancer death (25). However, additional studies will be needed to investigate the prognostic effect of biological age in oncology research. Besides, a nomogram was constructed as an objective instrument, which could assess the probability of 1- and 3-year survival for PDAC patients after radical surgery. The nomogram model contained four independent prognostic factors including age, reception of adjuvant chemotherapy, and AJCC 8th T and N stage. We first incorporated age into the nomogram model to predict prognosis of resected PDAC patients. The internal validation with the method of bootstrap was performed and showed an optimal conformity between actual and nomogram-predicted survival probability in the PDAC patient who received surgery.

Interestingly, subgroup analyses demonstrated that age remained its prognostic effect in PDAC patients with good nutritional status (normal albumin and hemoglobin) and relative low tumor burden (pancreatic body/tail cancer, N0 stage, without NI, normal AFP, CA125 and CA19-9, and without radiotherapy). These results were not surprising given the fact that among patients with low hemoglobin, albumin, white blood cell count, lymph node metastases, and elevated preoperative tumor marker, the aggressive cancer (26, 27) and poor nutritional status (28–32) would predominantly worsen the survival outcome, whereas the prognostic effect of aging was not apparent.

Our study also showed lower proportion of receiving adjuvant treatment in the elderly group (elderly vs. young, 68% vs. 83%). Meanwhile, adjuvant chemotherapy was the only independent prognostic factor among the elderly (HR=0.411; 95%CI, 0.201–0.837) and significantly improved OS of the elderly patients (mOS, no adjuvant chemotherapy vs. reception of adjuvant chemotherapy, 14.8 vs. 33.8 months). These were consistent with previous published studies. Nagrial et al. demonstrated that older patients (aged ≥70) were less likely to receive adjuvant chemotherapy (51.5% vs. 29.8%; p<0.0001). Older patients who did not receive adjuvant therapy was associated with worse OS (mOS, no adjuvant chemotherapy vs. reception of adjuvant chemotherapy, 13.1 vs. 21.8 months), and adjuvant chemotherapy is the only actionable variable associated with improved survival in older patients (33). The reason of less reception of adjuvant chemotherapy in the elderly patients could be attributed to the worse performance status (34), increased incidence of comorbidities (35), the perception of a less life expectancy, and the longer recovery time following surgery (36).

There were some limitations in our research. First, the study was a retrospective and single-center investigation. Our results require more prospective and multicenter studies for validation. Second, some clinical data such as specific chemotherapy regimens and postoperative complications were not included in this study. Postoperative complications were an important factor that could affect the decision on subsequent therapies. Taking different chemotherapy regimens could have discrepant prognosis. Then, the follow-up period was not long enough, and the median follow-up time was 40 months. As a result, the survival curves only showed 36-month survival time. Finally, there was only internal validation of the nomogram model. We did not perform external validation because of the low proportion of patients older than 70 years.

Taken together, our research indicated that elderly PDAC patients were associated with worse OS survival after radical resection, and the noticeable negative effect of aging was observed among PDAC patients with better preoperative nutritional status and less aggressive tumor biology. Adjuvant chemotherapy is essential to improve survival outcome of elderly PDAC patients after surgery.

## Data Availability Statement

The original contributions presented in the study are included in the article/**Supplementary Material**. Further inquiries can be directed to the corresponding authors.

## Ethics Statement

Written informed consent was obtained from the individual(s) for the publication of any potentially identifiable images or data included in this article.

## Author Contributions

YL, YM, WH, and DS contributed to conception and design of the study. SY organized the database. YM performed the statistical analysis. YM wrote the first draft of the manuscript. DY, XF, TT, and WC wrote sections of the manuscript. All authors contributed to the article and approved the submitted version.

## Conflict of Interest

The authors declare that the research was conducted in the absence of any commercial or financial relationships that could be construed as a potential conflict of interest.

## Publisher’s Note

All claims expressed in this article are solely those of the authors and do not necessarily represent those of their affiliated organizations, or those of the publisher, the editors and the reviewers. Any product that may be evaluated in this article, or claim that may be made by its manufacturer, is not guaranteed or endorsed by the publisher.
