# Supplementary material for: Prognostic Effect of Age in Resected Pancreatic Cancer Patients: A Propensity Score Matching Analysis
Source: Front Oncol. 2022 Mar 31;12:789351. doi: 10.3389/fonc.2022.789351 (PMC9008824; doi:10.3389/fonc.2022.789351)

Supplementary Material

| Supplemental table 1. Univariate and multivariate Cox regression analysis of the total cohort | | | | |
| --- | --- | --- | --- | --- |
|  | **Total cohort** | | | |
|  | **Univariate** | | **Multivariate** | |
|  | **HR** | **P-value** | **HR** | **P-value** |
| **Age (ref=age<70)** | 1.005(0.993-1.018) | 0.416 |  |  |
| **Sex (ref=male)** | 0.944(0.753-1.183) | 0.616 |  |  |
| **Tumor location**  **(ref=pancreatic head)** |  | **0.002** |  | 0.741 |
| **pancreatic body/tail** | 0.681(0.542-0.856) | 0.001 | 0.836(0.515-1.357) | 0.468 |
| **total pancreas** | 0.454(0.168-1.221) | 0.118 | 0.710(0.084-6.013) | 0.753 |
| **AJCC 8th T stage (ref=T1)** |  | **<0.001** |  | **0.062** |
| **T2** | 1.502(1.099-2.052) | 0.011 | 1.932(1.069-3.491) | 0.029 |
| **T3** | 1.818(1.247-2.651) | 0.002 | 1.893(0.935-3.836) | 0.076 |
| **T4** | 1.970(1.106-3.510) | 0.021 | 4.760(1.582-14.324) | 0.006 |
| **Tis** | 0.107(0.026-0.442) | 0.002 |  | 0.98 |
| **AJCC 8th N stage (ref=N0)** |  | **<0.001** |  | **0.05** |
| **N1** | 1.796(1.417-2.275) | <0.001 | 1.432(0.892-2.300) | 0.137 |
| **N2** | 3.548(2.431-5.177) | <0.001 | 2.331(1.150-4.725) | 0.019 |
| **Tumor differentiation**  **(ref=moderately-diff)** |  | **<0.001** |  | 0.193 |
| **poorly-diff** | 1.700(1.338-2.160) | <0.001 | 1.647(1.050-2.581) | 0.03 |
| **well-diff** | 0.083(0.020-0.336) | <0.001 |  | 0.978 |
| **un-diff** |  | 0.931 |  | 0.992 |
| **MVI (ref=no MVI)** | 1.321(0.985-1.772) | 0.063 |  |  |
| **FI (ref=no FI)** | 2.079(1.530-2.824) | **<0.001** | 1.625(0.857-3.081) | 0.137 |
| **NI (ref=no NI)** | 2.736(1.929-3.882) | **<0.001** | 1.075(0.502-2.305) | 0.852 |
| **Adjuvant chemotherapy**  **(ref=no-chemo)** | 0.510(0.394-0.661) | **<0.001** | 0.291(0.169-0.500) | **<0.001** |
| **Adjuvant radiotherapy**  **(ref=no-radio)** | 1.027(0.792-1.331) | 0.842 |  |  |
| **CA19-9 level (ref=CA19-9<35)** |  | **<0.001** |  | 0.74 |
| **CA19-9 35-200** | 1.527(1.115-2.093) | 0.008 | 0.902(0.437-1.861) | 0.78 |
| **CA19-9>200** | 1.923(1.414-2.616) | <0.001 | 0.620(0.159-2.417) | 0.491 |
| **Preglucose (continuous)** | 1.052(1.005-1.102) | **0.029** | 0.919(0.826-1.024) | 0.126 |
| **Albumin (continuous)** | 0.954(0.934-0.975) | **<0.001** | 0.995(0.935-1.059) | 0.87 |
| **Prealbumin (continuous)** | 0.054(0.008-0.357) | **0.002** | 1.049(0.010-115.713) | 0.984 |
| **Hemoglobin (continuous)** | 0.995(0.988-1.002) | 0.199 |  |  |
| **WBC (continuous)** | 0.962(0.907-1.019) | 0.189 |  |  |
| **Lymphocyte count (continuous)** | 0.813(0.666-0.993) | **0.043** | 1.101(0.748-1.620) | 0.627 |
| **Neutrophil count (continuous)** | 0.969(0.906-1.036) | 0.354 |  |  |
| **Monocyte count (continuous)** | 0.786(0.411-1.503) | 0.466 |  |  |
| **ASA (ref=grade1）** |  | 0.158 |  |  |
| **grade2** | 0.725(0.521-1.009) | 0.057 |  |  |
| **grade3** | 0.698(0.297-1.641) | 0.41 |  |  |
| **AFP (continuous)** | 1.041(0.979-1.108) | 0.201 |  |  |
| **CEA (continuous)** | 1.008(0.998-1.018) | 0.126 |  |  |
| **CA242 (continuous)** | 1.004(1.001-1.006) | **0.007** | 0.997(0.991-1.003) | 0.284 |
| **CA50 (continuous)** | 1.004(1.002-1.006) | **<0.001** | 1.008(1.001-1.016) | **0.034** |
| **CA125 (continuous)** | 1.003(0.999-1.007) | 0.101 |  |  |

Abbreviations: HR, hazard ratio; ref, reference; CA, Carbohydrate antigen; AJCC, American Joint Committee on Cancer; Tis, tumor in situ; MVI, microvascular invasion; FI, peripancreatic fat invasion; NI, neural invasion; WBC, white blood cell; ASA, American Society of Anesthesiologists; AFP, Alpha-fetoprotein; CEA, carcinoembryonic antigen

Supplemental Figure1. Overall survival Kaplan-Meier survival curves stratified by age of the total cohort.


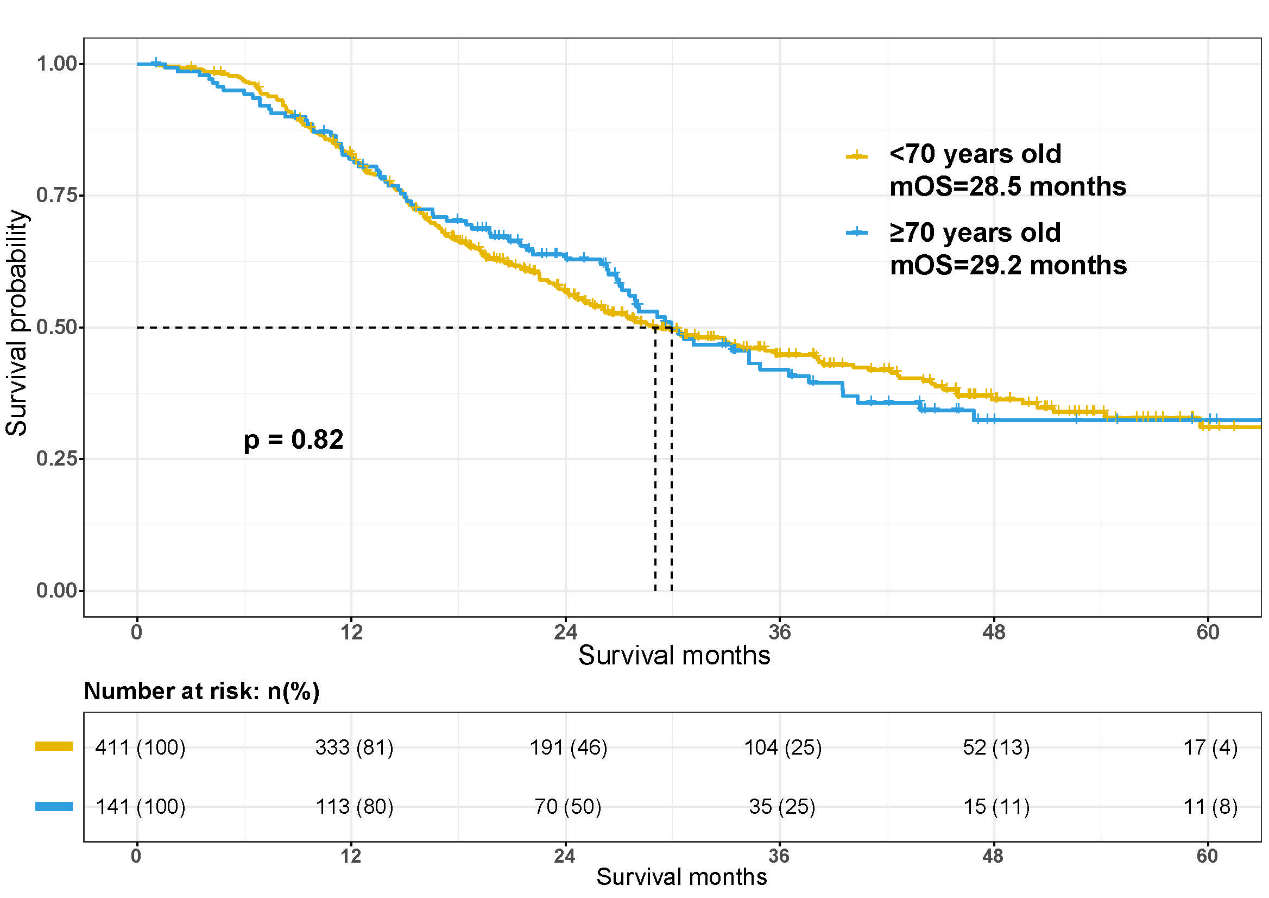

Supplement: Supplementary file 1 [file DataSheet_1.docx]
